# General features of existential depression in youth

**DOI:** 10.1192/j.eurpsy.2024.967

**Published:** 2024-08-27

**Authors:** V. Kaleda, U. Popovich

**Affiliations:** Mental Health Research Centre, Moscow, Russian Federation

## Abstract

**Introduction:**

The axial symptom of existential depression in youth is overvalued ideas about the meaninglessness of human life, its inconsistency with a certain “spiritual self-ideal”; ideas of humiliation, insolvency, low value, imperfection of society are noted, which accompanies varying degrees of severity of suicidal thoughts. The high suicidal risk, insufficient knowledge of such conditions makes it important to study.

**Objectives:**

Determination of the clinical and psychopathological consequences of existential depression in youth.

**Methods:**

53 male 16-25 years old with F31.3, F31.4, F32, F33 (ICD-10) with the existential depression were examined with clinical-psychopathological method, psychometric assessment of depression was carried out using the HDRS scale, assessment of suicidal intentions - using the C-SSRS scale. Also were analyzed: premorbid personality structure, hereditary burden in first-degree relatives, the role of exogenous provocations. Statistical data processing was carried out using the STATISTICA software package 10.0 for WINDOWS (StatSoft, USA, was used Pearson χ² test, Student t-test. The critical level of statistical significance is p ≤ 0.05.

**Results:**

A significant role in the manifestation of existential depression was played by exogenous provocations (χ²=9.47, p=0.05), especially psychotrauma: the most common were the death of a close relative or friend, unrequited love, and failure to enter the desired university.According to the premorbid personality structure schizoid (56.7%) and psychasthenic personalities (30.2%) prevailed. When assessing hereditary burden (χ²=9.59 p=0.047), pathocharacterological features were noted in first-degree relatives in 32.1% cases, affective disoders in 26, 4%.In terms of social and labor status (χ²=9.47, p=0.05), university students naturally predominated (56.6%). The average age of onset of depression was 17.8 ± 1.2 years, duration 3.7 ± 1.5 months. Non-suicidal self-harm was observed in 32.1%, especially in the initial stages of depression. Among suicidal tendencies (χ²=9.58, p=0.048), anti-vital thoughts (50.9%) and passive suicidal thoughts (34%) dominated; 5.7% of patients attempted suicide. On the HDRS scale, patients scored an average of 18.2±1.7 points, which reflected the severity of depression; the total score on the C-SSRS scale was 2.12±0.34.

**Conclusions:**

In the formation of existential depression, a significant role of exogenous provocations, especially psychotrauma, was discovered; a high suicidal risk was confirmed. Existential depressive states differed in duration; patients of the identified typological varieties scored high on the HDRS and C-SSRS scales. In the future, it is planned to study the follow-up group for the purpose of a detailed analysis of the dynamics of such conditions and their nosological affiliation.

**Disclosure of Interest:**

None Declared

